# Paradoxical long-term impact of maternal influenza infection on neonates and infants

**DOI:** 10.1186/s12879-020-05236-8

**Published:** 2020-07-11

**Authors:** Joon Young Song, Keon Vin Park, Sung Won Han, Min Joo Choi, Ji Yun Noh, Hee Jin Cheong, Woo Joo Kim, Min-Jeong Oh, Geum Joon Cho

**Affiliations:** 1 Division of Infectious Diseases, Department of Internal Medicine, Korea University College of Medicine, Seoul, Republic of Korea; 2 School of Industrial Management Engineering, Korea University, Seoul, Republic of Korea; 3 Department of Obstetrics and Gynecology, Korea University College of Medicine, Guro Hospital, Gurodong-ro 148, Guro-gu, Seoul, 08308 Republic of Korea

**Keywords:** Influenza, Pregnancy, Preterm birth, Low birth weight, Obesity

## Abstract

**Background:**

Pregnant women are at high risk of influenza-related morbidity and mortality. In addition, maternal influenza infection may lead to adverse birth outcomes. However, there is insufficient data on long-term impact of maternal influenza infection.

**Methods:**

This study was conducted to assess the impact of maternal influenza infection on birth outcomes and long-term influence on infants by merging the Korea National Health Insurance (KNHI) claims database and National Health Screening Program for Infants and Children (NHSP-IC). Mother-offspring pairs were categorized by maternal influenza infection based on the ICD-10 code.

**Results:**

Multivariate analysis revealed that maternal influenza infection significantly increased the risk of preterm birth (OR 1.408) and low birth weight (OR 1.198) irrespective of gestational age. The proportion of low birth weight neonates was significantly higher in influenza-infected women compared to those without influenza. However, since the fourth health screening (30–80 months after birth), the fraction of underweight was no longer different between children from influenza-infected and non-infected mothers, whereas the rates of overweight increased paradoxically in those born to mothers with influenza infection.

**Conclusions:**

Maternal influenza infection might have long-term effects on the health of children and adolescents even after infancy.

## Background

Physiological changes and immune adaptations occur during pregnancy to accommodate the fetus, resulting in impaired cell-mediated immunity [1]. These immune alterations impair pathogen clearance, and physiological adaptations including weight gain, increased cardiac output, decreased lung volume and hormonal changes increase the risk of severe infections in pregnant women [1, 2].

Pregnant women are at high risk of influenza-related morbidity and mortality. They are more likely to have severe infections and complications, leading to hospitalization and mortality. The mortality rate in pregnant women was high in the 1918, 1957 and 2009 influenza pandemics [3]. Although pregnant women represented only ≤1% of the population during the 2009 influenza H1N1 pandemic, they comprised 5% of all fatalities [4]. Among fatal cases, 7·1% occurred in the first trimester, 26·8% in the second trimester, and 64·3% in the third trimester [5]. Seasonal influenza infection also increases the risk of hospitalization, admission to an intensive care unit and death in pregnant women [6]. In the United Kingdom, pregnant women had an estimated four times greater mortality rate during a severe influenza season compared to a regular season [3]. In addition to the adverse maternal outcomes, maternal influenza infection may also lead to neonatal complications such as stillbirth, neonatal death, preterm delivery, and low birth weight, as shown during the 2009 H1N1 pandemic [7]. The World Health Organization (WHO) emphasizes pregnant women as the highest priority group for influenza vaccination and recommends that they should be vaccinated at any time of pregnancy since 2012 [8]. Nevertheless, only a limited number of countries (46%) worldwide have a national influenza immunization policy for pregnant women [9].

Despite increasing evidence supporting active influenza vaccination in pregnant women, some uncertainties remain. First, it is unclear whether influenza affects birth outcomes differently according to trimester of pregnancy. Second, further clarification is needed with regard to how long influenza-related adverse birth outcomes, particularly low birth weight might persist. The impact on low birth weight could vary depending on the stage of pregnancy at the time of influenza infection. The present study aims to assess the impact of maternal influenza infection on birth outcomes (preterm birth and low birth weight) stratified by pregnancy period, and the long-term influence on infants up to 80 months after birth.

## Methods

### Study population

This study was conducted by merging the Korea National Health Insurance (KNHI) claims database and National Health Screening Program for Infants and Children (NHSP-IC) [10, 11].

In Korea, 97% of the population is enrolled in the KNHI program. All claims information for these individuals is contained within the KNHI claims database. As part of the KNHI system, a National Health Screening Program for Infants and Children (NHSP-IC) was started in 2007 comprising seven consecutive health examinations based on age groups (4 to 9 months, 9 to 18 months, 18 to 30 months, 30 to 42 months, 42 to 54 months, 54 to 66 months, and 66 to 80 months). Data from this program, including physical examination, anthropometric examination, and developmental screening, are also contained within the database.

This study protocol was exempted for review by the Institutional Review Board of the Korea University Guro Hospital according to the exemption criteria.

### Dataset and study design

**Fig. 1:**
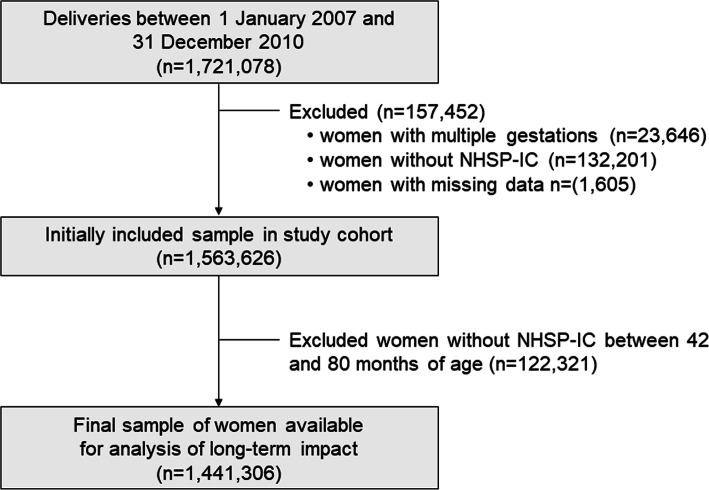
Flow Diagram of the Study Population

Figure 1 is a flowchart of study participant enrollment. To evaluate pregnancy outcomes using KNHI claims data, we identified all women who delivered singleton pregnancies between January 1, 2007 and December 31, 2010, and excluded women if their offspring did not undergo at least one of the seven consecutive NHSP-IC health examinations or had missing data (dataset 1). Offspring growth was assessed using body mass index (BMI) measurements taken between 42 and 80 months of age. For this analysis, we used KNHI claims data (dataset 1) in conjunction with the fourth through seventh NHSP-IC screening examination data (dataset 2).

Mother-offspring pairs were categorized by maternal influenza infection, and stratified by gestational age at influenza infection. Cases (mother-offspring pairs with maternal influenza infection) were compared to controls (mother-offspring pairs without maternal influenza infection) with respect to birth outcomes (preterm birth and low birth weight) and long-term influence on infants up to 80 months after birth.

### Definitions and measurement of outcomes

Maternal influenza infection during pregnancy was identified by a search for the relevant diagnosis codes in the International Classification of Diseases-10th Revision (ICD-10). Maternal influenza infection during pregnancy was defined if they were diagnosed with influenza (ICD-10 code J09, J10, and J11) from 280 days before delivery up to delivery. The gestational age at influenza infection was estimated retrospectively from the delivery: 181–280 days from delivery (1st trimester), 91–180 days from delivery (2nd trimester) and 0–90 days from delivery (3rd trimester). Pre-existing diabetes mellitus (both type 1 and 2) was defined as the recording of ICD-10 codes E10–14 at least two times per year, while pre-existing hypertension was defined as the presence of ICD-10 codes I10 or I11 at least two times per year.

Data regarding maternal and offspring outcomes were extracted from dataset 1. This information included pregnancy outcomes such as parity and delivery mode from KNHI claims data, and neonatal outcomes such as preterm birth, neonatal sex, and birthweight from the NHSP-IC database. Preterm birth was defined as gestational age < 37 weeks, low birth weight (LBW) was defined as birth weight < 2.5 kg, and macrosomia was defined as birth weight > 4.0 kg.

Offspring growth was analyzed using dataset 2. Current BMI was categorized according to age- and sex-specific BMI, which was derived from the NHSP-IC [11]. Underweight was defined as a BMI ≤ 10th percentile, while overweight was defined as a BMI ≥ 90th percentile.

### Statistical analysis

Continuous and categorical variables were expressed as mean ± standard deviation and percentages, respectively. Clinical characteristics were compared using Student’s t-test or ANOVA with Tukey’s post hoc test for continuous variables and the chi-square test for categorical variables. Multivariable logistic regression analysis was used to estimate the adjusted odds ratio (OR) and 95% confidence intervals (CIs) for the association of maternal influenza infection with adverse pregnancy outcomes and offspring growth. Generalized estimating equations (GEE) were used to evaluate the association between a maternal history of influenza infection and offspring growth longitudinally, considering the correlation between repeated measurements in the same individual. All tests were two-sided, and *p* < 0.05 was considered statistically significant. Statistical analyses were performed using SAS for Windows, version 9.4 (SAS Inc., Cary, NC, USA).

## Results

### Demographic and clinical characteristics of the study population

A total of 1,721,078 pregnant women delivered during the study period from 2007 to 2010 in South Korea according to KNHI records (Fig. 1). We excluded 23,646 women with multiple gestations, 132,201 women without NHSP-IC records and 1605 with missing values for the independent variables in the KNHI claims and NHSP-IC database (i.e., age, BMI, parity and birth weight). Initially, 1,563,626 mother-offspring pairs were included in the study cohort for analysis of the immediate impact of maternal influenza infection. As for the analysis of long-term impact, 122,321 pairs were further excluded because they did not undergo an NHSP-IC health examination between 42 and 80 months of age. Finally, 1,441,306 women were available for the analysis of long-term impact on the offspring, including 34,614 cases (pregnant women with influenza) and 1,406,692 controls without influenza. The baseline characteristics are described in Table 1 and Supplementary Table 1. The mean age was 30.3 years in influenza cases and 30.4 years in controls, and the proportion of advanced age mothers (≥35 years) was indistinguishable between the two groups irrespective of influenza infection (14.02% versus 13.78%, *p* = 0.1882). The prevalence of hypertension and diabetes was lower than 5% in both groups. More than 50% were primiparous women, and ~ 35% underwent cesarean delivery.

**Table 1:** Comparison of baseline characteristics and birth outcomes based on maternal influenza infection

Parameters		Controls without influenza (*n* = 1,525,972)	Cases with influenza (*n* = 37,654)	*p*-value
Maternal characteristics	Age, mean years ± SD	30.41 ± 3.81	30.31 ± 3.85	< 0.0001
	Advanced age with ≥35 years (n, %)	213,886 (14.02)	5188 (13.78)	0.1882
	Study year, n (%)			< 0.0001
	2007 year	377,086 (24.71)	9548 (25.36)	
	2008 year	380,237 (24.92)	7480 (19.87)	
	2009 year	372,847 (24.43)	7946 (21.10)	
	2010 year	395,802 (25.94)	12,680 (33.68)	
	Pre-pregnancy HTN (n, %)	43,443 (2.85)	1232 (2.76)	< 0.0001
	Pre-pregnancy DM (n, %)	60,406 (3.96)	1648 (4.38)	< 0.0001
	Primiparity (n, %)	805,385 (52.78)	18,930 (50.27)	< 0.0001
	Cesarean section (n, %)	539,328 (35.34)	13,680 (36.33)	< 0.0001
Birth outcomes	Neonatal sex–male (n, %)	787,549 (51.61)	19,428 (51.60)	0.9585
	Preterm birth (n, %)	40,798 (2.67)	1405 (3.73)	< 0.0001
	Birth weight (kg), mean ± SD	3.22 ± 0.49	3.20 ± 0.50	< 0.0001
	LBW (n, %)	56,773 (3.72)	1668 (4.43)	< 0.0001
	Macrosomia (n, %)	62,135 (4.07)	1532 (4.07)	0.9752

*SD* standard deviation; *HTN* hypertension; *DM* diabetes mellitus; *LBW* low birth weight

### Pregnancy period-stratified impact of maternal influenza infection on birth outcomes

As presented in Table 1, influenza-infected women were more likely to have preterm births (3.73% versus 2.67%, *p* < 0.0001) and deliver neonates with LBW (4.43% versus 3.72%, *p* < 0.0001) compared to the controls without influenza. Table 2 shows the pregnancy period-stratified impact of influenza infection on birth outcomes, including mean birth weight and the proportion of preterm births, LBW and macrosomia. Regardless of pregnancy period, maternal influenza infection increased the rates of preterm birth and LBW (*p* < 0.0001). On multivariate logistic regression analysis adjusting maternal age, diabetes, hypertension and primiparity, maternal influenza infection significantly increased the risk of preterm birth (first trimester, OR 1.500; second trimester, OR 1.190; third trimester, OR 1.432) and LBW (first trimester, OR 1.234; second trimester, OR 1.049; third trimester, OR 1.300) irrespective of pregnancy period, though the difference was statistically insignificant for LBW during the second trimester. There was no difference in the proportion of macrosomia between the two groups. When divided into the seasonal epidemic (2007–2008 years) and pandemic periods (2009–2010 years), the risk of preterm birth and LBW increased significantly among offspring with maternal influenza infection in both periods (Supplementary Tables 2 and 3).

**Table 2:** Adverse impact of maternal influenza infection on the birth outcomes, stratified by pregnancy periods

	Control (n = 1,525,972)	Cases with influenza – stratified by pregnancy periods at influenza infection			
		Trimester 1^†^ (*n* = 21,396)	Trimester 2^†^ (*n* = 9615)	Trimester 3^†^ (*n* = 6643)	*p*-value
Birth weight, mean ± SD (kg)^a^	3.216 ± 0.485^a^	3.203 ± 0.500^a^	3.209 ± 0.496^a^	3.194 ± 0.500^b^	< 0.0001
Preterm birth (n, %)^a^	40,798 (2.67) ^a^	847 (3.96) ^b^	305 (3.17) ^c^	253 (3.81) ^d^	< 0.0001
LBW (n, %)^a^	56,773 (3.72) ^a^	974 (4.55) ^b^	375 (3.90) ^c^	319 (4.80) ^d^	< 0.0001
Macrosomia (n, %)	62,135 (4.07)	848 (3.96)	404 (4.20)	280 (4.21)	0.7035
Logistic regression analysis					
**Preterm birth (n, %)**	unadjusted OR (95% CI)	age-adjusted OR (95% CI)	OR adjusted for age, diabetes, hypertension and primiparity (95% CI)		
Influenza (−)	ref	ref	ref		
Influenza at trimester 1	1.500 (1.400–1.608)	1.506 (1.405–1.614)	1.500 (1.399–1.607)		
Influenza at trimester 2	1.193 (1.064–1.337)	1.200 (1.070–1.346)	1.190 (1.062–1.335)		
Influenza at trimester 3	1.441 (1.271–1.635)	1.442 (1.271–1.636)	1.432 (1.262–1.625)		
Influenza at any trimester	1.411 (1.337–1.490)	1.416 (1.341–1.495)	1.408 (1.334–1.487)		
**LBW (n, %)**					
Influenza (−)	ref	ref	ref		
Influenza at trimester 1	1.234 (1.157–1.317)	1.238 (1.160–1.321)	1.234 (1.156–1.316)		
Influenza at trimester 2	1.050 (0.947–1.165)	1.055 (0.951–1.170)	1.049 (0.945–1.163)		
Influenza at trimester 3	1.306 (1.167–1.462)	1.307 (1.167–1.463)	1.300 (1.161–1.455)		
Influenza at any trimester	1.200 (1.141–1.261)	1.203 (1.145–1.264)	1.198 (1.140–1.259)		
**Macrosomia (n, %)**					
Influenza (−)	ref	ref	ref		
Influenza at trimester 1	0.973 (0.908–1.042)	0.975 (0.910–1.044)	0.973 (0.908–1.043)		
Influenza at trimester 2	1.034 (0.936–1.142)	1.038 (0.939–1.147)	1.036 (0.937–1.144)		
Influenza at trimester 3	1.037 (0.920–1.169)	1.037 (0.920–1.169)	1.035 (0.918–1.166)		
Influenza at any trimester	1.000 (0.950–1.053)	1.002 (0.951–1.055)	1.000 (0.949–1.053)		

^a^ The same letters indicate non-significant differences between groups based on Tukey’s multiple comparison test and Chi-square test

^†^ Trimester 1 = 181–280 days from delivery; trimester 2 = 91–180 days from delivery; trimester 3 = 0–90 days from delivery

*SD* standard deviation; *LBW* low birth weight

### Long-term impact of maternal influenza infection on offspring

The long-term impact of maternal influenza infection was evaluated with respect to birth outcomes using national health screening data for infants and children up to 80 months after birth (Table 3). Although the proportion of underweight infants was significantly higher initially in neonates from influenza-infected women (case group), the fraction of underweight children was no longer different between the two groups from the fourth national health screening (30–42 months after birth) onward, and became lower among the case group at the fifth and sixth screenings (42–66 months after birth). Conversely, the rates of overweight increased paradoxically in children born to mothers with influenza infection. On multivariate analysis adjusting for maternal age, diabetes, hypertension, primiparity, cesarean section, preterm labor, birth weight and neonatal sex, offspring with maternal influenza infection were at significantly higher risk of being overweight at the fourth (30–42 months after birth, OR 1.057) and seventh (66–80 months after birth, OR 1.063) health screenings (Table 3). Similarly, on GEE analysis assuming within-subject correlation, offspring from influenza-infected mothers were no longer underweight, and more likely to be overweight (adjusted OR 1.039, 95% CI 1.007–1.071) compared to those from mothers without influenza at 30–80 weeks after birth (Table 4). When divided into the seasonal epidemic (2007–2008 years) and pandemic periods (2009–2010 years), the risk of being overweight increased significantly among offspring with maternal influenza infection in both periods (Supplementary Table 4).

**Table 3:** Long-term impact of maternal influenza infection on the offspring: multivariate logistic regression analysis

Infant health screening	Offspring born to control (*n* = 1,406,692)	Offspring born to women with a history of influenza (*n* = 34,614)	*p*-value	Unadjusted OR (95% CI)	Adjusted OR^a^ (95% CI)
Underweight, No. (%)					
4th (30–42 months)	97,312/979,890 (9.9%)	2351/24,358 (9.7%)	0.1502	0.969 (0.928–1.012)	0.966 (0.925–1.009)
5th (42–54 months)	69,982/953,954 (7.3%)	1656/23,592 (7.0%)	0.0652	0.954 (0.907–1.004)	0.948 (0.901–0.998)
6th (54–66 months)	65,354/886,721 (7.4%)	1489/21,609 (6.9%)	0.0076	0.930 (0.882–0.981)	0.926 (0.877–0.976)
7th (66–80 months)	51,921/624,845 (8.3%)	1134/14,144 (8.0%)	0.2135	0.962 (0.905–1.023)	0.956 (0.899–1.017)
Overweight, No. (%)					
4th (30–42 months)	77,412/979,890 (7.9%)	2031/24,358 (8.3%)	0.0123	1.060 (1.013–1.110)	1.057 (1.009–1.107)
5th (42–54 months)	113,147/953,954 (11.9%)	2854/23,592 (12.1%)	0.2672	1.023 (0.983–1.064)	1.020 (0.980–1.061)
6th (54–66 months)	111,473/886,721 (12.6%)	2780/21,609 (12.9%)	0.1984	1.027 (0.986–1.069)	1.025 (0.984–1.067)
7th (66–80 months)	82,126/624,845 (13.1%)	1969/14,144 (13.9%)	0.0068	1.069 (1.018–1.121)	1.063 (1.013–1.116)
BMI (kg/m^2^), mean (SD)					
4th (30–42 months)	16.0942 (1.4050)	16.1306 (1.4357)	< 0.0001		
5th (42–54 months)	16.0248 (1.4412)	16.0456 (1.4382)	0.0281		
6th (54–66 months)	15.9905 (1.6017)	16.0198 (1.6687)	0.0109		
7th (66–80 months)	16.1291 (1.8858)	16.1709 (1.8875)	0.0091		

^a^ Multivariate logistic regression analysis was performed. ORs were adjusted for maternal age, diabetes, hypertension, primiparity, cesarean section, preterm labor, birth weight and neonatal sex

*OR* odds ratio; *CI* confidence interval

**Table 4:** Long-term impact of maternal influenza infection on the offspring: repeated measures analysis by generalized estimating equations

	Unadjusted OR (95% CI)	Adjusted OR^a^ (95% CI)
Overweight	1.043 (1.011–1.075)	1.039 (1.007–1.071)
Underweight	0.951 (0.918–0.985)	0.948 (0.915–0.981)

Repeated measures analysis was performed using generalized estimating equations (GEE)

^a^ ORs were adjusted for maternal age, diabetes, hypertension, primiparity, cesarean section, preterm labor, birth weight and neonatal sex

*OR* odds ratio; *CI* confidence interval

## Discussion

This study provides meaningful results by assessing the long-term impact of maternal influenza infection during pregnancy on newborns. First, this study shows that maternal influenza infection might cause preterm birth and LBW irrespective of pregnancy period. Second, paradoxically during long-term follow-up, influenza infection during pregnancy increased the risk of obesity (overweight) in children. The short-term impacts of maternal influenza infection on birth outcomes have been evaluated, mainly focusing on preterm birth, LBW or small for gestational age (SGA) [12, 13]. According to a recent systematic review, the impact of maternal influenza infection on birth outcomes seems to be closely related to disease severity [12]. As for preterm birth, heterogeneity was noted across more than ten studies [12]. However, increased preterm birth risk was reported after severe maternal influenza infection in two studies during the 2009 H1N1 influenza pandemic (odds ratio, 2.39 and 4.00) and in another two studies during seasonal epidemics (odds ratio, 3.82 and 4.08) [7, 14–16]. Mothers with influenza infection had a higher risk of having LBW infants in a meta-analysis (odds ratio 1.71, 95% CI 1.03–2.84) [13]. Although a small number of studies have been conducted with inconsistent results regarding SGA, two studies showed a statistically significant correlation between maternal influenza infection and SGA with odds ratios of 1.59 (95% CI, 1.15–2.20) and 1.66 (95% CI, 1.11–2.49) [17, 18]. In addition, another study reported increased risk of SGA after maternal influenza infection (adjusted odds ratio 2.35, 95% CI 1.03–5.36) when confined to severe disease [19].

Although many observational studies have been performed to evaluate the immediate birth outcomes from maternal influenza infection, long-term prognosis has not yet been assessed [12, 13]. In this study, the BMIs of newborns born to influenza-infected and non-infected mothers were compared up to 80 months after birth. Although newborns of influenza-infected mothers were more likely to have LBW, a higher proportion of them were unexpectedly overweight during long-term follow-up. It is unclear why this phenomenon occurred and how it will carry forward into adulthood. Nevertheless, we have several hypotheses. First, our results might be explained in part by the ‘thrifty phenotype hypothesis,’ which refers to the notion that fetal malnutrition may cause adult chronic diseases [20, 21]. In an environment where a pregnant mother cannot get enough nutrition and catabolism is increased, the fetus will preferentially divert nutrients to essential organs, such as the brain, to survive. An organ such as the pancreas, which was undernourished in fetus stage, remains immature, resulting in the necessity for newborns to get more nutrients in a compensatory manner after birth. Second, there is the possibility that parents may be more attentive to nutrition when a newborn is born small. It is possible that these mechanisms underlie adult chronic diseases including diabetes and obesity. Long-term follow-up studies from childhood to adulthood are required to test our hypotheses.

Four large clinical trials have been conducted to evaluate the effect of maternal influenza immunization in Bangladesh, South Africa, Mali and Nepal [22–25]. In the earlier trials carried out in Bangladesh, South Africa and Mali, the focus was on the assessment of vaccine efficacy against laboratory-confirmed influenza in infants under 6 months old through maternal immunization [22, 24, 25]. In those trials, maternal influenza vaccination consistently had good efficacy in preventing influenza infection among infants: Bangladesh 63% (95% CI, 5–85), South Africa 49% (95% CI, 12–70) and Mali 33% (95% CI, 4–54) [22, 24, 25]. The most recent trial in Nepal showed a significant reduction in LBW births (15, 95% CI 3–25) through maternal influenza immunization in addition to the prevention of laboratory-confirmed influenza in infants younger than 6 months (30, 95% CI 5–48) [23]. Numerous observational studies have been conducted to assess influenza vaccine effectiveness in preventing adverse birth outcomes [26, 27]. A meta-analysis showed that the seasonal influenza vaccine is effective in preventing LBW (26, 95% CI 12–39), consistent with the results of the randomized clinical trial in Nepal [27]. In addition, maternal influenza vaccination reduced the likelihood of stillbirth (relative risk 0.73, 95% CI 0.55–0.96) in the prior meta-analysis, but did not have significant preventive effects on spontaneous abortion (relative risk 0.91, 95% CI 0.68–1.22) [26].

This study has some limitations. First, we did not consider the influenza vaccination history of pregnant women or the severity of influenza infection. However, the study period (2007–2010 years) was largely a pre-maternal vaccination era, so maternal influenza vaccination rate might have been quite low throughout this time period except perhaps during the 2009 H1N1 pandemic. In the future, if the influenza vaccination of the pregnant woman is included in the national immunization program and vaccination data becomes available, further analysis will be possible. Second, data on some potential confounders (breastfeeding, childhood nutrition, and physical activity, socioeconomic status) were not available in this study. Finally, due to limitations of the data source, influenza virus subtype-dependent differences in the effect on birth outcomes could not be assessed.

The major strength of this study is the large sample size of mother-offspring pairs, and being able to compare adverse birth outcomes over consecutive influenza seasons, including a major pandemic. We assessed the impact of maternal influenza infection on newborns longitudinally for a long period after birth (up to 80 months). Furthermore, the long-term follow-up data were collected by well-trained investigators using the same protocol.

## Conclusions

This study shows that influenza infection is associated with preterm birth and LBW in newborns and may paradoxically increase the risk of obesity in the long-term. Maternal influenza infection might have long-term effects on the health of children and adolescents even after infancy. These findings suggest maternal vaccination with influenza may potentially protect against low birthweight in babies, which may then in turn prevent obesity in children and across the lifespan. This has the potential for huge health and economic consequences.

## Supplementary information

**Additional file 1 Table S1.** Comparison of baseline characteristics and birth outcomes based on maternal influenza infection during seasonal epidemic and pandemic periods. **Table S2.** Adverse impact of maternal influenza infection on the birth outcomes during seasonal epidemic (2007–2008 years), stratified by pregnancy periods. **Table S3.** Adverse impact of maternal influenza infection on the birth outcomes during pandemic period (2009–2010 years), stratified by pregnancy periods. **Table S4.** Long-term impact of maternal influenza infection on the offspring: subgroup analysis (seasonal epidemic and pandemic periods)

## Data Availability

The data that support the findings of this study are available from the National Health Insurance Service (NHIS), but restrictions apply to the availability of these data, which were used under license for the current study and so are not publicly available. Data are however available from the authors upon reasonable request and with permission of the NHIS. The results do not necessarily represent the opinion of the National Health Insurance Corporation.

## Abbreviations

WHO: World Health Organization
KNHI: Korea National Health Insurance
NHSP-IC: National Health Screening Program for Infants and Children
BMI: Body mass index
ICD-10: INTERNATIONAL Classification of Diseases-10th Revision
LBW: Low birth weight
OR: Odds ratio
GEE: Generalized estimating eqs.
CI: Confidence interval
SGA: Small for gestational age
SD: Standard deviation
HTN: Hypertension
DM: Diabetes mellitus
