# Supplementary material for: Paradoxical long-term impact of maternal influenza infection on neonates and infants
Source: BMC Infect Dis. 2020 Jul 11;20:502. doi: 10.1186/s12879-020-05236-8 (PMC7353799; doi:10.1186/s12879-020-05236-8)
Supplement: Supplementary file 1 — Additional file 1 Table S1. Comparison of baseline characteristics and birth outcomes based on maternal influenza infection during seasonal epidemic and pandemic periods. Table S2. Adverse impact of maternal influenza infection on the birth outcomes during seasonal epidemic (2007–2008 years), stratified by pregnancy periods. Table S3. Adverse impact of maternal influenza infection on the birth outcomes during pandemic period (2009–2010 years), stratified by pregnancy periods. Table S4. Long-term impact of maternal influenza infection on the offspring: subgroup analysis (seasonal epidemic and pandemic periods) [file 12879_2020_5236_MOESM1_ESM.docx]

**Supplementary Table 1.** Comparison of baseline characteristics and birth outcomes based on maternal influenza infection during seasonal epidemic and pandemic periods

| **Study periods** | **Parameters** | | **Controls without influenza** | **Cases with influenza** | ***p*-value** |
| --- | --- | --- | --- | --- | --- |
| **Seasonal epidemic periods (2007-2008 years)** | **Maternal characteristics** | No. of subjects | 757,323 | 17,028 |  |
|  |  | Age, mean years ± SD | 30.15 ± 3.80 | 30.15 ± 3.88 | <0.0001 |
|  |  | Advanced age with ≥35 years (n, %) | 94,821 (12.52) | 2,192 (12.87) | 0.1696 |
|  |  | Pre-pregnancy HTN (n, %) | 19,121 (2.52) | 525 (3.08) | <0.0001 |
|  |  | Pre-pregnancy DM (n, %) | 26,700 (3.53) | 661 (3.88) | 0.0128 |
|  |  | Primiparity (n, %) | 407,531 (53.81) | 8,976 (52.71) | 0.0045 |
|  |  | Cesarean section (n, %) | 267,984 (35.39) | 6,304 (37.02) | <0.0001 |
|  | **Birth outcomes** | Neonatal sex–male (n, %) | 390,504 (51.56) | 8,852 (51.98) | 0.2767 |
|  |  | Preterm birth (n, %) | 19,603 (2.59) | 657 (3.86) | <0.0001 |
|  |  | Birth weight (kg), mean ± SD | 3.22 ± 0.49 | 3.21 ± 0.50 | <0.0001 |
|  |  | LBW (n, %) | 27,915 (3.69) | 792 (4.65) | <0.0001 |
|  |  | LGA (n, %) | 32,260 (4.26) | 768 (4.51) | 0.1097 |
| **Pandemic periods (2009-2010 years)** | **Maternal characteristics** | No. of subjects | 768,649 | 20,626 |  |
|  |  | Age, mean years ± SD | 30.68 ± 3.81 | 30.45 ± 3.82 | <0.0001 |
|  |  | Advanced age with ≥35 years (n, %) | 119,065 (15.49) | 2,996 (14.53) | 0.0002 |
|  |  | Pre-pregnancy HTN (n, %) | 24,322 (3.16) | 707 (3.43) | 0.0331 |
|  |  | Pre-pregnancy DM (n, %) | 33,706 (4.39) | 987 (4.79) | 0.0057 |
|  |  | Primiparity (n, %) | 397,854 (51.76) | 9,954 (48.26) | <0.0001 |
|  |  | Cesarean section (n, %) | 271,344 (35.30) | 7,376 (35.76) | 0.1732 |
|  | Birth outcomes | Neonatal sex–male (n, %) | 397,045 (51.65) | 10,576 (51.28) | 0.2814 |
|  |  | Preterm birth (n, %) | 21,195 (2.76) | 748 (3.63) | <0.0001 |
|  |  | Birth weight (kg), mean ± SD | 3.21 ± 0.48 | 3.20 ± 0.50 | 0.0009 |
|  |  | LBW (n, %) | 28,858 (3.75) | 876 (4.25) | 0.0002 |
|  |  | LGA (n, %) | 29,875 (3.89) | 764 (3.70) | 0.1803 |

Abbreviation: SD, standard deviation; HTN, hypertension; DM, diabetes mellitus; LBW, low birth weight; LGA, large for gestational age.

**Supplementary Table 2.** Adverse impact of maternal influenza infection on the birth outcomes during seasonal epidemic (2007-2008 years), stratified by pregnancy periods

|  | **Control (n=757,323)** | **Cases with influenza – stratified by pregnancy periods** | | | |
| --- | --- | --- | --- | --- | --- |
|  |  | **Trimester 1^b^ (n =10,790)** | **Trimester 2^b^ (n =3,715)** | **Trimester 3^b^ (n =2,523)** | ***p*-value** |
| Birth weight, mean ± SD (kg)^a^ | 3.221 (0.486) ^a^ | 3.209 (0.500) ^a, b^ | 3.215 (0.516) ^a, b^ | 3.197 (0.485) ^b^ | 0.0052 |
| Preterm birth (n, %)^a^ | 19,603 (2.59) ^a^ | 437 (4.05) ^b^ | 128 (3.45) ^b^ | 92 (3.65) ^b^ | <0.0001 |
| LBW (n, %)^a^ | 27,915 (3.69) ^a^ | 503 (4.66) ^b^ | 159 (4.28) ^c^ | 130 (5.15) ^c^ | <0.0001 |
| LGA (n, %) | 32,260 (4.26) | 482 (4.47) | 166 (4.47) | 120 (4.76) | 0.3920 |
|  |  |  |  |  |  |
| Logistic regression analysis |  |  |  |  |  |
| **Preterm birth (n, %)** | unadjusted OR (95% CI) | age-adjusted OR (95% CI) | OR adjusted for age, diabetes, hypertension and primiparity (95% CI) | | |
| Influenza (-) | ref | ref | ref |  |  |
| Influenza at trimester 1 | 1.589 (1.442-1.750) | 1.588 (1.442-1.750) | 1.581 (1.435-1.742) |  |  |
| Influenza at trimester 2 | 1.343 (1.125-1.603) | 1.349 (1.130-1.610) | 1.338 (1.121-1.598) |  |  |
| Influenza at trimester 3 | 1.424 (1.156-1.755) | 1.416 (1.149-1.744) | 1.403 (1.139-1.729) |  |  |
| Influenza at any trimester | 1.510 (1.395-1.635) | 1.510 (1.395-1.635) | 1.501 (1.387-1.625) |  |  |
| **LBW (n, %)** |  |  |  |  |  |
| Influenza (-) | ref | ref | ref |  |  |
| Influenza at trimester 1 | 1.278 (1.168-1.399) | 1.278 (1.168-1.399) | 1.273 (1.163-1.393) |  |  |
| Influenza at trimester 2 | 1.168 (0.996-1.370) | 1.172 (1.000-1.375) | 1.165 (0.993-1.366) |  |  |
| Influenza at trimester 3 | 1.422 (1.191-1.697) | 1.416 (1.186-1.690) | 1.403 (1.176-1.675) |  |  |
| Influenza at any trimester | 1.275 (1.186-1.371) | 1.275 (1.186-1.371) | 1.268 (1.180-1.364) |  |  |
|  |  |  |  |  |  |
| **LGA (n, %)** |  |  |  |  |  |
| Influenza (-) | ref | ref | ref |  |  |
| Influenza at trimester 1 | 1.051 (0.959-1.152) | 1.051 (0.958-1.152) | 1.049 (0.957-1.151) |  |  |
| Influenza at trimester 2 | 1.051 (0.899-1.229) | 1.055 (0.902-1.233) | 1.052 (0.900-1.229) |  |  |
| Influenza at trimester 3 | 1.122 (0.934-1.349) | 1.118 (0.930-1.343) | 1.116 (0.928-1.341) |  |  |
| Influenza at any trimester | 1.062 (0.987-1.142) | 1.062 (0.987-1.142) | 1.060 (0.985-1.140) |  |  |

Abbreviation: SD, standard deviation; LBW, low birth weight; LGA, large for gestational age.

^a^ The same letters indicate non-significant differences between groups based on Tukey's multiple comparison test and Chi-square test

^b^ Trimester 1 = 181-280 days from delivery; trimester 2 = 91-180 days from delivery; trimester 3 = 0-90 days from delivery

**Supplementary Table 3.** Adverse impact of maternal influenza infection on the birth outcomes during pandemic period (2009-2010 years), stratified by pregnancy periods

|  | **Control (n=768,649)** | **Cases with influenza – stratified by pregnancy periods** | | | |
| --- | --- | --- | --- | --- | --- |
|  |  | **Trimester 1^b^ (n =10,606)** | **Trimester 2^b^ (n =5,900)** | **Trimester 3^b^ (n =4,120)** | ***p*-value** |
| Birth weight, mean ± SD (kg)^a^ | 3.211 (0.484) ^a^ | 3.198 (0.500) ^a, b^ | 3.205 (0.482) ^a, b^ | 3.193 (0.509) ^b^ | 0.0041 |
| Preterm birth (n, %)^a^ | 21,195 (2.76) ^a^ | 410 (3.87) ^b^ | 177 (3.00) ^a^ | 161 (3.91) ^b^ | <0.0001 |
| LBW (n, %)^a^ | 28,858 (3.75) ^a^ | 471 (4.44) ^b^ | 216 (3.66) ^a^ | 189 (4.59) ^b^ | <0.0001 |
| LGA (n, %) | 29,875 (3.89) | 366 (3.45) | 238 (4.03) | 160 (3.88) | 0.1275 |
|  |  |  |  |  |  |
| Logistic regression analysis |  |  |  |  |  |
| **Preterm birth (n, %)** | unadjusted OR (95% CI) | age-adjusted OR (95% CI) | OR adjusted for age, diabetes, hypertension and primiparity (95% CI) | | |
| Influenza (-) | Ref | ref | ref |  |  |
| Influenza at trimester 1 | 1.418 (1.284-1.567) | 1.427 (1.292-1.577) | 1.422 (1.287-1.571) |  |  |
| Influenza at trimester 2 | 1.091 (0.939-1.267) | 1.102 (0.948-1.280) | 1.094 (0.942-1.272) |  |  |
| Influenza at trimester 3 | 1.434 (1.224-1.680) | 1.445 (1.233-1.692) | 1.438 (1.228-1.685) |  |  |
| Influenza at any trimester | 1.328 (1.233-1.430) | 1.338 (1.243-1.442) | 1.331 (1.236-1.434) |  |  |
| **LBW (n, %)** |  |  |  |  |  |
| Influenza (-) | ref | ref | ref |  |  |
| Influenza at trimester 1 | 1.191 (1.086-1.308) | 1.198 (1.091-1.315) | 1.195 (1.088-1.311) |  |  |
| Influenza at trimester 2 | 0.974 (0.850-1.117) | 0.982 (0.857-1.125) | 0.977 (0.852-1.120) |  |  |
| Influenza at trimester 3 | 1.233 (1.065-1.427) | 1.240 (1.071-1.435) | 1.236 (1.068-1.431) |  |  |
| Influenza at any trimester | 1.137 (1.062-1.218) | 1.144 (1.068-1.225) | 1.140 (1.064-1.221) |  |  |
| **LGA (n, %)** |  |  |  |  |  |
| Influenza (-) | ref | ref | ref |  |  |
| Influenza at trimester 1 | 0.884 (0.796-0.982) | 0.889 (0.800-0.987) | 0.886 (0.798-0.984) |  |  |
| Influenza at trimester 2 | 1.039 (0.913-1.184) | 1.048 (0.920-1.194) | 1.046 (0.919-1.192) |  |  |
| Influenza at trimester 3 | 0.999 (0.853-1.171) | 1.005 (0.858-1.178) | 1.003 (0.856-1.175) |  |  |
| Influenza at any trimester | 0.952 (0.885-1.024) | 0.957 (0.890-1.030) | 0.955 (0.888-1.028) |  |  |

Abbreviation: SD, standard deviation; LBW, low birth weight; LGA, large for gestational age.

^a^ The same letters indicate non-significant differences between groups based on Tukey's multiple comparison test and Chi-square test

^b^ Trimester 1 = 181-280 days from delivery; trimester 2 = 91-180 days from delivery; trimester 3 = 0-90 days from delivery

**Supplementary Table 4.** Long-term impact of maternal influenza infection on the offspring: subgroup analysis (seasonal epidemic and pandemic periods)

|  | **Infant health screening** | **Offspring born to control** | **Offspring born to women with a history of influenza** | ***p*-value** | **Unadjusted OR**  **(95% CI)** | **Adjusted OR^a^**  **(95% CI)** |
| --- | --- | --- | --- | --- | --- | --- |
| **Seasonal epidemic periods (2007-2008 years)** | Underweight, No. (%) |  |  |  |  |  |
|  | 4th (30-42 months) | 43,323/429,351 (10.1%) | 937/9,620 (9.7%) | 0.2592 | 0.962 (0.899-1.030) | 0.959 (0.896-1.027) |
|  | 5th (42-54 months) | 30,275/416,576 (7.3%) | 624/9,290 (6.7%) | 0.0430 | 0.919 (0.846-0.997) | 0.915 (0.843-0.994) |
|  | 6th (54-66 months) | 28,244/396,295 (7.1%) | 572/8,638 (6.6%) | 0.0709 | 0.924 (0.848-1.007) | 0.920 (0.844-1.003) |
|  | 7th (66-80 months) | 31,370/384,099 (8.2%) | 600/8,244 (7.3%) | 0.0035 | 0.883 (0.812-0.960) | 0.879 (0.808-0.956) |
|  | Overweight, No. (%) |  |  |  |  |  |
|  | 4th (30-42 months) | 36,005/429,351 (8.4%) | 848/9,620 (8.8%) | 0.1334 | 1.057 (0.985-1.135) | 1.055 (0.982-1.133) |
|  | 5th (42-54 months) | 50,689/416,576 (12.2%) | 1,162/9,290 (12.5%) | 0.3215 | 1.032 (0.970-1.099) | 1.028 (0.965-1.094) |
|  | 6th (54-66 months) | 50,656/396,295 (12.8%) | 1,126/8,638 (13.0%) | 0.4860 | 1.023 (0.960-1.090) | 1.017 (0.954-1.084) |
|  | 7th (66-80 months) | 50,592/384,099 (13.2%) | 1,159/8,244 (14.1%) | 0.0185 | 1.078 (1.013-1.148) | 1.070 (1.004-1.139) |
|  | BMI, mean (SD) |  |  |  |  |  |
|  | 4th (30-42 months) | 16.0943 (1.4397) | 16.1312 (1.4380) | 0.0129 |  |  |
|  | 5th (42-54 months) | 16.0369 (1.5158) | 16.0767 (1.5333) | 0.0124 |  |  |
|  | 6th (54-66 months) | 16.0117 (1.6518) | 16.0612 (1.8153) | 0.0119 |  |  |
|  | 7th (66-80 months) | 16.1394 (1.9016) | 16.2163 (1.8592) | 0.0002 |  |  |
| **Pandemic periods (2009-2010 years)** | Underweight, No. (%) |  |  |  |  |  |
|  | 4th (30-42 months) | 53,989/550,539 (9.8%) | 1,414/14,738 (9.6%) | 0.3922 | 0.976 (0.924-1.032) | 0.974 (0.921-1.030) |
|  | 5th (42-54 months) | 39,707/537,378 (7.4%) | 1,032/14,302 (7.2%) | 0.4343 | 0.975 (0.914-1.040) | 0.969 (0.908-1.034) |
|  | 6th (54-66 months) | 37,110/490,426 (7.6%) | 917/12,971 (7.1%) | 0.0344 | 0.929 (0.868-0.995) | 0.925 (0.864-0.991) |
|  | 7th (66-80 months) | 20,551/240,746 (8.5%) | 534/5,900 (9.1%) | 0.1626 | 1.066 (0.974-1.167) | 1.058 (0.967-1.159) |
|  | Overweight, No. (%) |  |  |  |  |  |
|  | 4th (30-42 months) | 41,407/550,539 (7.5%) | 1,183/14,738 (8.0%) | 0.0217 | 1.073 (1.010-1.140) | 1.067 (1.004-1.133) |
|  | 5th (42-54 months) | 62,458/537,378 (11.6%) | 1,692/14,302 (11.8%) | 0.4442 | 1.020 (0.969-1.074) | 1.017 (0.966-1.072) |
|  | 6th (54-66 months) | 60,817/490,426 (12.4%) | 1,654/12,971 (12.8%) | 0.2318 | 1.033 (0.980-1.088) | 1.033 (0.980-1.089) |
|  | 7th (66-80 months) | 31,534/240,746 (13.1%) | 810/5,900 (13.7%) | 0.0369 | 1.062 (1.002-1.128) | 1.060 (1.001-1.123) |
|  | BMI, mean (SD) |  |  |  |  |  |
|  | 4th (30-42 months) | 16.0941 (1.3773) | 16.1302 (1.4343) | 0.0026 |  |  |
|  | 5th (42-54 months) | 16.0154 (1.3804) | 16.0255 (1.3726) | 0.3889 |  |  |
|  | 6th (54-66 months) | 15.9734 (1.5599) | 15.9921 (1.5628) | 0.1782 |  |  |
|  | 7th (66-80 months) | 16.1126 (1.8602) | 16.1075 (1.9249) | 0.8394 |  |  |

Abbreviation: OR, odds ratio; LBW, low birth weight; SD, standard deviation.

^a^ Adjusted for maternal age, diabetes, hypertension, primiparity, cesarean section, preterm labor, birth weight and neonatal sex
